# Efficient Development of Green Emulsifier/Emollient-Based Emulsion Vehicles: From RSM Optimal Experimental Design to Abridged In Vivo Assessment

**DOI:** 10.3390/pharmaceutics15020486

**Published:** 2023-02-01

**Authors:** Mila Vukašinović, Sanela Savić, Nebojša Cekić, Tanja Ilić, Ivana Pantelić, Snežana D. Savić

**Affiliations:** 1 Department of Pharmaceutical Technology and Cosmetology, University of Belgrade-Faculty of Pharmacy, Vojvode Stepe 450, 11221 Belgrade, Serbia; 2 DCP Hemigal, R&D Sector, Tekstilna 97, 16000 Leskovac, Serbia; 3 Faculty of Technology, University of Niš, Bulevar Oslobodjenja 124, 16000 Leskovac, Serbia

**Keywords:** Lauryl Glucoside, Myristyl Glucoside and Polyglyceryl-6 Laurate, C15–19 Alkane, design of experiments, rheology, texture analysis, skin friction

## Abstract

Since natural-origin, sustainable ingredients are preferred by modern consumers, novel emulsifiers and emollients keep entering the market. This study hypothesizes that a combination of in silico, instrumental tools and simplified sensory studies could be used to efficiently characterize emulsions in a shorter timeframe. A total of 22 rather simple o/w emulsions were prepared by a time/energy-saving emulsification process. A natural mixed emulsifier (Lauryl Glucoside/Myristyl Glucoside/Polyglyceryl-6 Laurate) and two emollients (both with INCI name C15–19 Alkane) were used. The performed D-optimal experimental design within the response surface method (RSM) significantly narrowed down the number of samples about to enter the stage of texture, friction and sensory studies to the samples comprising 30% of a respective Emogreen emollient and 2% or 3% of the emulsifier. The sample comprising 2% emulsifier/30% Emogreen^®^ L15 showed significantly higher firmness (42.12 mN) when compared to the one with 2% emulsifier/30% Emogreen^®^ L19 (33.62 mN), which was somewhat unexpected considering the emollients’ inherent viscosity values (4.5 mPa·s for L15 and 9 mPa·s for L19). The sample with 2% emulsifier/30% Emogreen^®^ L19 managed to maintain the lowest friction, while the one with 3% emulsifier/30% Emogreen^®^ L19 released its full lubricating potential in the second part of the measurement (30–60 s). The obtained results revealed the strengths and weaknesses of each formulation, narrowing down their possible applications in the early development stage.

## 1. Introduction

The concept of sustainability has become a growing challenge for many fields of industries, including cosmetics and pharmaceutical ones. There is a strong market tendency to develop more sustainable products using ‘green’, environmentally friendly raw materials but, at the same time, it is a difficult task to successfully replace conventional high-performing synthetic ingredients with sustainable natural alternatives in order to maintain product quality, stability, safety and effectiveness [1,2]. This substitution may be quite challenging because of the instability and performance and aesthetic limitations linked with the use of natural ingredients [1]. Whatever the purpose of a final formulation may be, the expectations of a modern consumer should not be neglected because they are important factors for the sale potential of a cosmetic product [2]. Modern patients sometimes show a similar frame of mind. Therefore, some findings obtained during cosmetic formulation development prove to be also valuable for pharmaceutical topical products, particularly in the case of emollients intended for atopic and itchy skin treatment [3,4].

In cosmetics, emulsion systems are leading formulations and optimal vehicles for active ingredients [5]. The reason for the vast use of oil in water (o/w) emulsions in skin care products, particularly low viscosity o/w emulsions (lotions), is that they have a high water content (implying lower cost); fresh, light, less greasy feel; and good skin spreadability [6]. Emollients are essential ingredients of topical emulsions, responsible for smoother, more elastic and lubricated skin as well as for a pleasant skin feel [7]. Furthermore, emollients can hydrate the skin by a (semi)occlusive effect, which lowers transepidermal water loss (TEWL) [8]. Emollient oils may also significantly impact the emulsions’ consistency and spreadability [7]. These factors determine the appropriate efficiency and user acceptance of a product [1,7]. Natural, plant-based and sustainable emollients are preferred by modern consumers and may even improve patient adherence in chronic dermatoses maintenance [4]. However, conventional ones are often prone to oxidation and hence highly unstable [9]. Considering the sustainability trend, it is also desirable to use biodegradable and mild, so-called eco- and skin-friendly, emulsifiers in order to obtain stable emulsions [10]. Alkylpolyglucosides (APGs) are commonly defined as a class of natural, carbohydrate-derived surfactants, produced from renewable resources [10]. They represent an established group of highly effective nonionic surfactants, having attractive interfacial properties. APGs are ecologically safe and polyethylene glycol (PEG)-free and thus often referred to as green surfactants [11]. Unsurprisingly, new APG representatives are constantly being developed.

Emulsions have to meet numerous demands and expectations regarding stability, safety, efficacy (both real and perceived) and adequate aesthetic (visual perception) and sensory properties in order to be eligible to users [12,13]. Especially for the assessment of stability and sensory properties, rheology, textural, tribological and sensory evaluation are crucial. A combination of in silico, instrumental tools and simplified sensory studies could be used to characterize an emulsion in order to obtain appropriate results in a shorter timeframe with less expense. 

During formulation development, in order to obtain a high-quality emulsion product with desired physicochemical features, sensorial properties and efficacy, different factors (formulation and processing) should be taken into consideration, preferably simultaneously. This may significantly lengthen the entire process. In this sense, the ‘design of experiments’ (DoE) approach may be implemented, with the aim to identify and manage [14,15] the way the critical quality attributes of the developed model emulsions are influenced by variations of emulsion composition, namely emulsifier concentration, emollient concentration and emollient type, and their interactions, with the final goal of ensuring the desired product performance.

D-optimal experimental design within the response surface method (RSM) relies on specific computer algorithms, moving away from the standard, orthogonal-like ones and enabling the correlation of the effects of the tested variables. The main benefit of the D-optimal RSM lies in the fact that it can potentially fit a range of models (e.g., first order, second order, quadratic and cubic) with a smaller number of supporting experimental studies. Hence, it is not surprising that D-optimal design is frequently selected by the cosmetic and pharmaceutical industries, especially when formulating emulsion systems—from nanoemulsions to creams [16,17,18].

Further, rheological characterization is essential for the formulation development process and quality control of cosmetic products and helps to elucidate the effects of ingredient composition and interaction on the final formulation properties. It is well known that the application and acceptance of emulsions largely depend on the flow properties of the final product [13]. Continual (steady-state) rheological measurements are usually carried out to predict product behavior in real-time conditions during manufacture and application [19]. The parameters obtained from these measurements (different viscosity values, yield stress and hysteresis loop area value) are linked to certain sensory attributes and provide information about the systems’ colloidal structure, which strongly influences the sensory characteristics and long-term stability [12,13]. Besides rheological measurements, textural analysis is another classic technique for evaluating emulsion performance, but it is even more useful when predicting the sensory properties of the product, as sensorial perceptions stem from the textural properties of the formulation [20]. Considering that consumer testing is often expensive and time consuming, there is much interest to set up instrumental techniques to objectively quantify certain attributes of formulations [6].

However, instruments can miss some subjective aspects despite the fact that they provide quantitative information and accurately measure product characteristics, so sensory studies should supplement rheology and texture analysis [21]. For this reason, subjective surveys of cosmetic products are standard practice because there is a critical need for direct consumer feedback [22]. Ideally, cosmetic products should perfectly meet the sensory expectations of consumers, who usually select a skin care product for its function and promising efficacy but are mostly seduced by the pleasantness it brings [23]. In consumer sensory studies, the assessors evaluate the intensity of product attributes or the reaction of a particular body area [22]. The results of the assessment, correlating instrumental measurements, can be a very effective tool for predicting consumer responses. The skin feel during and after product application is an important criterion for consumer acceptance [24]. Some undesirable topical product characteristics as communicated by consumers are, for instance, ‘difficult to spread’, ‘sticky’, ‘too long to be absorbed’, ‘too greasy’ and ‘leaving too much residue’ [25]. To achieve adequate efficacy and user acceptance of a cosmetic emulsion, spreading is an important property; it can be influenced by the choice of ingredients, in particular of emollients [26,27,28].

In order to apply all points that were previously discussed about trends in emulsion development and characterization, in the present study, 22 rather simple o/w lotions (fluid emulsion systems) were prepared by the cold emulsification process (time- and energy-saving, greener method). For emulsion stabilization, a new, natural, cold processable and sustainable emulsifier was used, comprising two APGs and one polyglyceryl ester. It allows the development of products with desirable sensory attributes (light and pleasant after-feel) [29]. As emollients, three different nonpolar oils were varied, including traditional light liquid paraffin and two novel sustainable emollients belonging to the class of C15-C19 alkanes, promoted as biodegradable sensory alternatives to silicone oils (good spreading, nonsticky touch and matte effect) [29]. The type and concentration of a single-component oil phase and the concentration of the mixed emulsifier were varied in order to compare the physicochemical, sensorial, textural and tribological (frictional) features of the developed emulsions. During formulation development, in order to obtain emulsions with the required properties, a scientific and systemic design of experiments (DoE) was applied. This approach allowed for determining, predicting and controlling the manner in which the critical rheological attributes of developed emulsions are affected by variations in emulsion composition [5]. The final objective was to select formulations with desired, optimized rheological performance, stability and sensorial properties.

## 2. Materials and Methods

### 2.1. Materials

A key emulsion stabilizer (mixed emulsifier comprising Lauryl Glucoside, Myristyl Glucoside and Polyglyceryl-6 Laurate) named Fluidifeel^®^ Easy was kindly provided by Seppic (Paris, France), along with the varied biocompatible emollients Emogreen^®^ L15 and Emogreen^®^ L19 (both attributed with the same International Nomenclature of Cosmetic Ingredients (INCI) name, C15–19 Alkane). Paraffinum liquidum and Glycerin were purchased from Fagron (Trikala, Greece). A preservative mixture consisting of Ethylhexylglycerin and Phenoxyethanol (Sharomix^®^ EG 10) was obtained from Sharon Laboratories (Ashdod, Israel), while Xanthan Gum (Safic Care^®^ T XGC 80) was from Safic-Alcan (Milano, Italy). Pharmaceutical-grade purified water was obtained in-house using a Gen Pure Ultrapure device (Thermo Fisher Scientific GmbH, Munich, Germany).

### 2.2. Preparation of the Samples

All emulsions (precise composition given in Table 1), differing in type and/or concentration of emollients and emulsifier, were prepared by a ‘cold’ emulsification process (at room temperature). Being in liquid form, the nonionic O/W emulsifier (2% or 3% (*w*/*w*) of lauryl glucoside/myristyl glucoside/polyglyceryl-6 laurate mixture) was added to the aqueous phase, followed by stirring with a rotor-stator homogenizer (Ultra-Turrax T25, IKA, Staufen, Germany) at 6000 rpm for 40 s. Subsequently, the oil phase consisting of 10%, 20% or 30% (*w*/*w*) of a single emollient (light liquid paraffin, Emogreen^®^ L15 or Emogreen^®^ L19) was slowly added to the aqueous phase while stirring at 4400 rpm. The obtained emulsion was then homogenized for 5 min at 8000 rpm. In the last minute of the homogenization, 1% (*w*/*w*) of the preservative was added. Finally, the emulsion was transferred to a laboratory stirrer with a propeller mixing tool (Heidolph RZR 2020, Heidolph, Schwabach, Germany) and proceeded with the addition of the rheology modifier (xanthan gum dispersed in glycerin) at 650 rpm for 6 min.

### 2.3. DoE—Response Surface D-Optimal Experimental Design

To identify and evaluate the simultaneous influence of key formulation variables on critical quality attributes of the developed model emulsions, a computer-generated D-optimal experimental design within the RSM was applied. Given the authors’ experience in formulating emulsion-based products, the concentration of emulsifier along with the concentration and type of emollient was determined as the main formulation factors that can typically affect the critical physicochemical properties, specifically rheological performances of the developed emulsions, and, therefore, their overall acceptability. According to the employed design, a total of 22 experimental runs (14 model points, 4 lack-of-fit points and 4 replicate points) were generated and randomly performed. As the response variables, rheological parameters (apparent viscosity, hysteresis area, elastic and viscous moduli and yield point), pH value and electrical conductivity of the designed model emulsions were determined. The factor-level combinations, experimental plan and the responses of each experimental run are given in Table 1.

By applying the response surface regression procedure, the experimentally acquired data were analyzed to fit an adequate polynomial model (linear, two-factor interaction and quadratic) that could best describe the evaluated response in relation to the investigated factors. For design planning, data processing and statistical analysis (analysis of variance, ANOVA), Design–Expert^®^ software (version 11.1.0; Stat-Ease Inc., Minneapolis, MN, USA) was employed. The best predictive models for the investigated responses were proposed based on the significant model terms (factor coefficients, *p* < 0.05), insignificant lack of fit (*p* > 0.1), maximized values for the multiple correlation coefficient (R-squared, R^2^) and adjusted R^2^, as well as the reasonable agreement of the predicted R^2^ with the adjusted R^2^ (the difference < 0.2), all provided by Design–Expert^®^. To find the optimum levels for the tested factors leading to the emulsions with desired rheological performances, the optimization procedure was performed. For easier interpretation of the factor effects, three-dimensional (3D) surface plots of the evaluated responses were also constructed where appropriate.

### 2.4. Light Microscopy

A preview of the investigated samples’ microstructure was enabled by the Olympus BX53 microscope (Olympus Europa Holding GmbH, Hamburg, Germany), equipped with CellSens Entry 1.15 software. The samples were observed in triplicate using a 10× magnification, after which the representative micrographs were captured. The process was repeated after 3 months (samples stored at room temperature) for the sake of a preliminary stability check.

### 2.5. pH and Conductivity Measurement

The pH measurements were performed by immersing the previously calibrated potentiometric probe of a pH meter (Hanna Instruments Inc. 8417, Woonsocket, RI, USA) directly into the samples. The conductivity of each emulsion was measured using a conductivity meter (CDM 230, Radiometer, Brønshøj, Denmark). Conductometry is commonly used to determine the type of emulsion and monitor its stability over time. This method is sensitive to small changes in the emulsion structure. Both tests were performed in triplicate for each sample, after which the mean values and standard deviations were calculated. The measurements were repeated after 3 months of storage at room temperature.

### 2.6. Rheological Characterization

The rheological behavior of the developed model emulsions was analyzed with an MCR 302 air-bearing rheometer (Anton Paar, Graz, Austria), equipped with coaxial cylinders system CC27/C-PTD 200 (including appropriate evaporation blocker system) and driven by RheoCompass™ software (also Anton Paar). Continuous rotational tests were captured at 20 °C within the shear rate ranges of 0.1–100 s^−1^ (upward curve) and 100–0.1 s^−1^ (downward curve); the parameters acquired were apparent viscosity (interpolated at a shear rate of 50 s^−1^) and hysteresis area. Dynamical mechanical analysis (oscillatory tests) included amplitude sweeps performed at radial frequency value (ω) of 10 rad s^−1^ in the deformation range of 0.01–100%. The parameters obtained from amplitude sweeps were yield point (yield stress and yield), determined as the shear stress value at the limit of nondestructive, reversible elastic deformations’ region (linear viscoelastic (LVE) region) (linearity limit); storage (elastic) modulus G′; and loss (viscous) modulus G″ [30].

### 2.7. Texture Analysis

The prepared emulsions were analyzed with EZ-LX Texture analyzer (Shimadzu, Kyoto, Japan). The immersion/deimmersion test (2-cycle compression analysis) was applied with two varying measurement tools: 1.3 cm diameter cylindrical aluminum jig and 3 cm diameter conical aluminum press jig. The test speed was set to 1 mm/s and the immersion distance to 30 mm. All measurements were carried out in triplicate at a constant temperature of 20 ± 2 °C. The built-in Trapezium-X Single software was used for data analysis. The measurements were reassessed after 3 months.

### 2.8. Skin Friction Assessment

The following two studies (2.7. and 2.8.) were performed after obtaining permission from the Ethical Committee of the Faculty of Pharmacy, University of Belgrade, Serbia (approval number: 1137/2) and written informed consent from the involved volunteers. During the tribological study, 5 healthy female volunteers aged 28 ± 4 years and without previous history or clinical signs of skin conditions were instructed to refrain from washing their arms or using any product 24 h before the beginning of the study. Volunteers were asked to rest for at least 30 min prior to the measurements, in controlled room conditions (temperature 22 ± 1 °C and relative humidity 40 ± 5%).

The skin friction coefficient (expressed as arbitrary Frictiometer^®^ units by the accompanying MPA software) was determined using Frictiometer^®^ FR700 (Courage + Khazaka, Köln, Germany) equipped with a smooth Teflon (PTFE) disk. Friction measurements were performed for each of the 22 samples at a constant speed of 90 rpm for 60 s (one measurement taken per second). A quantity of 5.5 μL cm^−2^ of each sample was applied with a micropipette on the volar side of forearms using a cardboard ruler. One defined site per volunteer served as the nontreated control (NC).

### 2.9. Sensory Study

With the aim of obtaining real-life feedback, an abridged sensory study similar to the one performed by Calixto et al. [31] was performed with the same 5 female volunteers. A day before the actual study, the panelists were provided with clear written instructions on how to perform this descriptive test using the given intensity scales. During the training, panelists were provided with testers belonging to the low, middle or high score on the intensity scale. Seven sensory descriptors in total were defined in order to evaluate the formulations before, throughout and after application:

- Viscosity = malleability of the formulation in the container and upon first application (scores 0 to 100);
- Cohesiveness/elasticity = degree of a sample’s deformation/stretching when applied and separated between two fingers; 100 μL of each sample was applied to the thumb (score 0 = no filaments and score 100 = the sample stretches in filaments > 2 cm long);
- Spreadability = easiness of a sample’s application over the skin surface (score 0 = extremely hard and score 100 = extremely easy);
- Stickiness = relative force required to separate the fingers from the skin surface upon a sample’s application. In 3.5 cm diameter circles, 30 µL of each sample was rubbed in using 10 identical circular movements over 7 s. After 3 min, the tested skin area was touched with a clean finger and the stickiness of the residual film was estimated (score 0 = slightly sticky and score 100 = extremely sticky);
- Absorption = easiness of sample uptake by the skin (score 0 = nonabsorptive and score 100 = very easily absorbed);
- Residual film = the amount of the film left on the skin 10 min after sample application (score 0 = no apparent film and score 100 = thick residual film);
- Skin hydration = relative impression of skin hydration 3 min after a sample’s application (score 0 = does not hydrate the skin and score 100 = marked skin hydration feel).

During the analysis of the results, sensory descriptors relevant to the samples’ texture profiling were discerned.

### 2.10. Statistical Analysis

Where applicable, data were presented as mean SD. The results of the characterization of the tested FFS were statistically compared using Student’s *t*-test or one-way analysis of variance (ANOVA). The results of the in vivo skin performance study were either analyzed by Student’s *t*-test or ANOVA followed by the Tukey post hoc test or by the nonparametric Kruskal–Wallis test followed by the Wilcoxon signed-rank test or Mann–Whitney U test for pairwise comparisons between groups. Normality of data was assessed using the Shapiro–Wilk test. Statistical analysis was performed using PASW Statistics software package, version 18.0 (SPSS Inc., Chicago, IL, USA). The level of significance was set to *p* < 0.05.

## 3. Results and Discussion

The C15–19 alkanes investigated within this study belong to a new generation of emollients produced by a patented production process (so-called Total Special Fluids patented technology). This original, solvent-free method results in high-purity liquid alkanes that are readily biodegradable in seawater (80% and 83% biodegradability found for Emogreen^®^ L15 and L19, respectively, while following OECD protocol 306) [32,33,34]. These are plant-based alkanes that are NATRUE-, COSMOS- and ECOCERT-certified and derived from responsible plant biomass [32]. Envisioned as alternatives to volatile silicone oils, these natural emollients have a neutral CO_2_ footprint when measured “from cradle to gate”, whereas the production of silicones requires five times more energy [32]. Emogreen^®^ L15 and L19 are inert, nonpolar oils with the following properties revealed by the producer: viscosity at 20 °C—4.5 mPa·s and 9 mPa·s; refractive index at 20 °C—1.433 and 1.434; specific gravity—0.775 and 0.785; flash point—105 °C and 135 °C; pour point—−65 °C and −30 °C; vapor pressure at 20 °C—0.002 kPa and 0.001 kPa; and contact angle value at 0.8 s (20 °C)—3.3 and 4.7, for L15 and L19, respectively [34]. Since their application potential spans from skin care, hair care, hygiene and makeup to sun care, the producer provides a rather wide recommended concentration range, from 0.5 to 50%. 

Fluidifeel^TM^ Easy is a natural, O/W and nonionic emulsifier suitable for low-viscous, sprayable formulations. Within the recommended use of 1–3%, it allegedly emulsifies a range of oil types (esters, mineral, vegetable, silicone oils, etc.) and tolerates the presence of electrolytes well, optimally between pH 4 and 8 [35]. However, the most prominent feature of this mixed emulsifier is its suitability for both hot and cold preparation/manufacturing processes [35].

Taking into consideration the data available for the investigated emulsifier and emollients so far, our study design started with the D-optimal experimental design within the response surface method.

### 3.1. RSM Optimal Experimental Design

During formulation development of fluid O/W emulsions stabilized by the novel glycolipid emulsifier and containing emollients with different polarities, the RSM optimal experimental design (D-criterion) was utilized to analyze, systematically and simultaneously, the impact of three main formulation factors, namely the concentration of emulsifier (varied at two levels) and concentration and type of emollient (varied at three levels each), on the critical performances of the developed model emulsions. The employed design strategy thus enabled us to identify and quantify not only significant single factors but also significant factor interactions, which are not possible to detect with the traditional one factor at a time method. This approach was already successfully employed for developing some emulsion-based formulations [36,37,38]. According to the built design matrix (Table 1), a total of 22 emulsions were prepared by the cold emulsification method and comprehensively characterized in terms of their physicochemical properties, namely rheological behavior, electrical conductivity and pH value.

**Figure 1:**
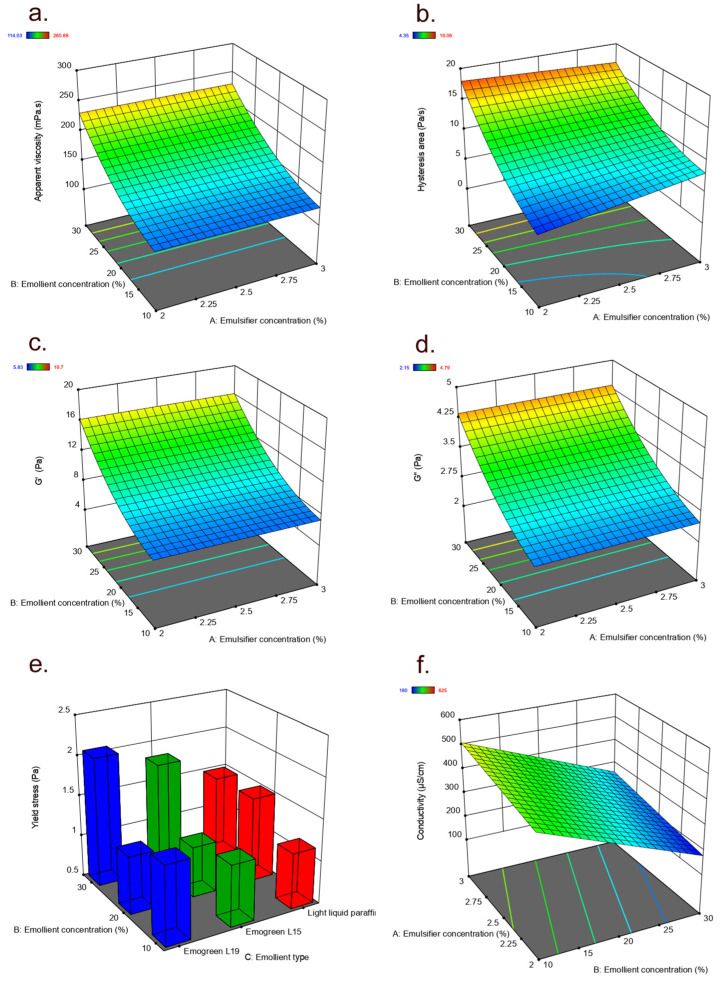
Response surface plots for the apparent viscosity (**a**), hysteresis area (**b**), storage modulus G′ (**c**), loss modulus G″ (**d**), yield point (**e**) and electrical conductivity (**f**) of the developed green emulsions as a function of the experimental formulation factors—emulsifier concentration (A), emollient concentration (B) and emollient type (C).

For each measured response (Table 1), the direct effect of each individual variable (emulsifier concentration and emollient concentration and type), as well as the interaction effect, was calculated and their statistical significance was checked. A *p*-value less than 0.05 indicated that the model term (factor/interaction) had a significant effect on the investigated response. Insignificant model terms (*p* > 0.1) were removed from the proposed models (except those required to support hierarchy), and thus the final reduced models for the estimated parameters were created. The resulting response equations in terms of coded factors and statistical analysis of the proposed models are given in Table 2, while the graphical representations of the fitted models are shown in Figure 1 in the form of 3D surface plots.

The ANOVA statistics showed that the generated models for all investigated rheological parameters as well as for pH value and electrical conductivity were significant (*p* < 0.05 for the model F value; Table 2), suggesting that the listed responses (except yield point and pH value) could be well described by the proposed models. Furthermore, the fitted models had adequate R^2^, adjusted R^2^, predicted R^2^ and precision values (not counting those for yield point and pH value; Table 2), further supporting their use for the approximative prediction of the listed responses (within the actual experimental region). The experimental design results were further analyzed, focusing on the relative impact of the model factors by comparing their corresponding coefficients; the higher the magnitude of each coefficient in the coded equation, the higher the respective factor effect on the investigated response. In addition, a positive sign for the factor coefficient indicates a synergistic effect, while a negative sign indicates an antagonistic effect of the corresponding factor on the evaluated response [5]. 

### 3.2. Rheological Characterization

**Figure 2:**
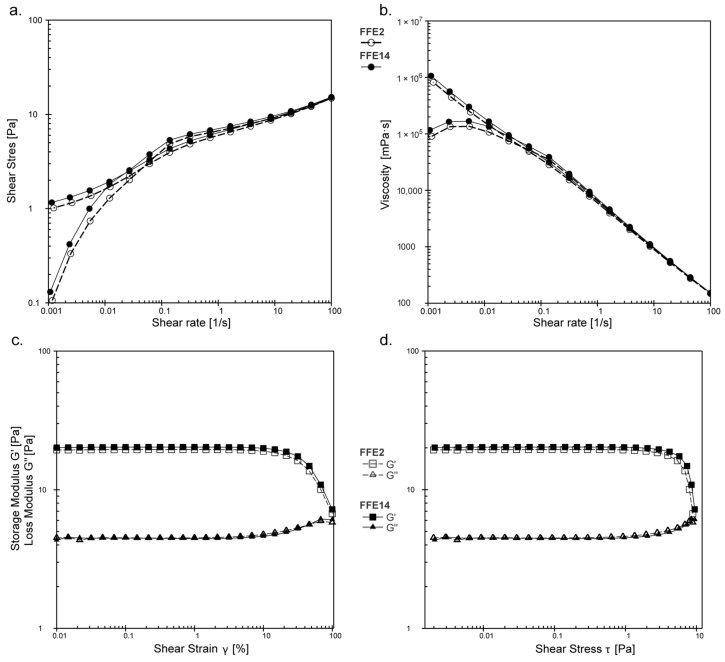
Rheological properties of the developed emulsions: flow curves (**a**); viscosity curves (**b**); amplitude sweeps—storage and loss moduli vs. shear strain (**c**) and shear stress (**d**) for two selected model formulations (FFE2 and FFE14).

Flow and viscosity curves acquired from continuous rotational tests (Figure 2a,b) showed that all prepared model emulsions exhibited a pseudoplastic (shear thinning) flow behavior, with the apparent viscosity values ranging from 114 to 261 mPa s and the hysteresis area values in the range of 4.4–19.1 Pa s^−1^ (Table 1). This type of flow behavior could be considered favored for most topically applied products, since it makes product application easier, including pouring the product out of the packaging as well as spreading on the skin [39]. Furthermore, all prepared emulsions were characterized by dominating elastic behavior (G′ values ranged from 5.8 to 19.7 Pa; Table 1) over viscous behavior (G″ values in the range of 2.2 to 4.8 Pa; Table 1), i.e., showed the character of viscoelastic solids (G′ (reflects the solid character of the sample) > G″ (reflects the liquid character of the sample)) in the LVE region of amplitude sweeps (oscillatory tests) (Figure 2c,d), further suggesting certain physical stability at relative state at rest [30]. In addition, all emulsion formulations revealed a certain yield point (0.7–2.6 Pa; Table 1), which was determined as the shear stress value at the limit of the LVE range, i.e., the shear stress value beyond which the system starts to flow.

The experimental design results (Table 2) confirmed that the differences observed in the rheological behavior of the developed model emulsions could be attributed to the differences in emulsion composition. However, considering apparent viscosity as well as elastic and viscous moduli (G′ and G″, respectively) from Table 2, only the emollient concentration (B) was found to be significant and also positive, imposing a nonlinear (squared term, B2) increasing effect on these rheological properties of the developed emulsion samples. In other words, increasing the emollient content from 10% to 20% and further to 30% generally yielded emulsions with higher apparent viscosity and higher G′ and G″ values (Figure 1a,c,d, respectively), irrespective of the emulsifier concentration or emollient type in the formulation. The exception was the emulsion prepared with Emogreen^®^ L15 as emollient and stabilized with 3% of the emulsifier, where increasing the concentration of emollient from 10% to 20% resulted in lower apparent viscosity and G′ and G″ values, whereas a further increase in the emollient to 30% produced emulsions with higher values of these responses. This discrepancy stems from a combination of Emogreen^®^ L15′s inherent properties and the fundamental mixing principles of rotor-stator devices. Namely, the lower intrinsic viscosity (4.5 mPa·s) and contact angle value (3.3) of this emollient make it more sensitive to processing parameters such as mixing time and rotor speed, thus affecting the total interfacial area. As a result, depending on the critical quality attributes (in this case, the amount of the emollient) and critical processing parameters, the oil’s brake-up into a population of droplets during this turbulent flow may sometimes occur simultaneously, and sometimes in sequence, resulting in different rheological parameters [40]. In this manner, it appears that Emogreen^®^ L15 shares some advantages but also certain disadvantages with silicone oils, since low-viscosity silicone oils are sometimes known for unpredictable droplet breakage during rotor-stator processing [41].

The ANOVA results (Table 2) further revealed that the hysteresis area as a measure of thixotropy of the developed emulsions was significantly influenced not only by the emollient concentration alone (nonlinear positive effect, B2) but also its combined effect with emulsifier concentration (AB interaction). Looking closely at the surface plot (Figure 1b), it could be noticed that in the case of model emulsions stabilized with either 2% or 3% of the emulsifier, increasing the emollient concentration from 10% to 20% and further to 30% resulted in a higher hysteresis area. On the other hand, when the content of the emulsifier was changed from 2% to 3%, a tendency of hysteresis area increase was shown in emulsions prepared with 10% and 20% of the emollient, contrary to emulsions containing 30% of the emollient, where the hysteresis area tended to decrease (Figure 1b). This is again in line with the previously discussed challenges of high-shear mixing and suggests that certain emulsifier: emollient ratios would require preparation process optimization.

Concerning the yield point, regarded as an indicator of emulsion internal structure at rest and long-term storage stability, the statistics in Table 2 show that only the emollient concentration (B) could have a significant effect (despite the accompanying low R^2^, the predicted R² was in reasonable agreement with the adjusted R^2^). In addition, although no significant interactions were observed, some trends in yield point could still be noticed with variation in formulation composition. By analyzing the 3D plot (Figure 1e), it could be further seen that a change in the emollient content from 10% to 20% correlated with increases in the yield values of model emulsions prepared with light liquid paraffin, whereas a slight decrease could be observed in emulsions containing either Emogreen^®^ L15 or Emogreen^®^ L19 as emollients, stabilized with either 2% or 3% of the emulsifier. However, further increasing the emollient Emogreen^®^ L15 or Emogreen^®^ L19 to 30% gave emulsions with higher yields, regardless of the emulsifier concentration, while almost no difference was observed in the yield values of the model emulsions containing light liquid paraffin as the emollient (Figure 1e). This supports the position of liquid paraffin as a common and robust oil phase for topical emulsions (although its mineral origin is unpopular in some consumer groups) and is based on the already established behavior of liquid paraffin droplets. Namely, upon dispersion, liquid paraffin tends to form droplets with low deformability (sometimes referred to as ‘hard’ droplets). This was noted for a range of liquid paraffin concentrations and a variety of emulsifiers [42]. As opposed to liquid paraffin, the investigated alkanes tend to form ‘soft’ droplets.

### 3.3. Light Microscopy, pH Value and Electrical Conductivity Measurements

**Figure 3:**
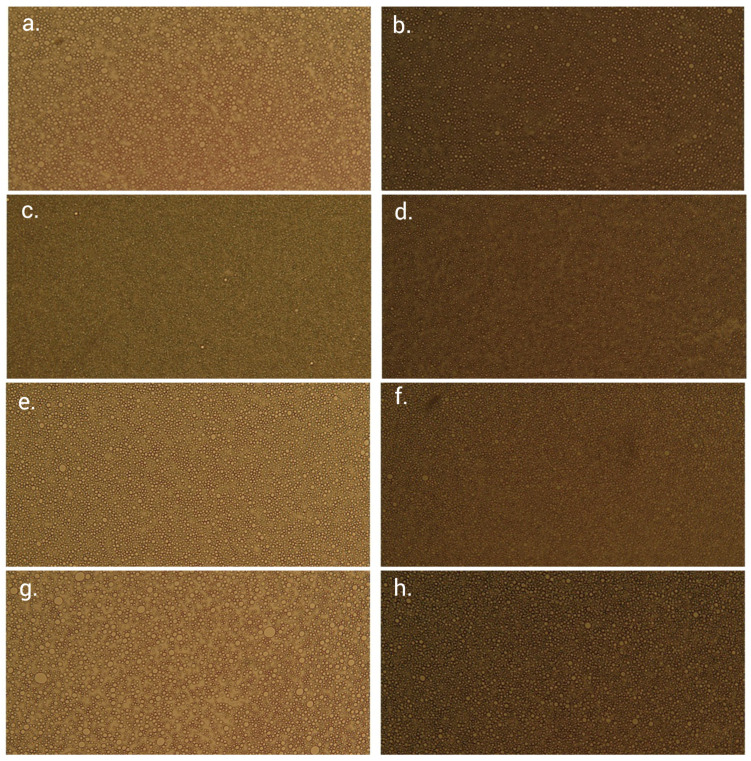
Representative micrographs captured with 10× magnification, allowing insights into the investigated samples’ microstructure upon preparation (**a**) FFE2, (**c**) FFE9, (**e**) FFE14 and (**g**) FFE16 and after 90 days of storage at room temperature (**b**) FFE2, (**d**) FFE9, (**f**) FFE14 and (**h**) FFE16.

Monitoring an emulsion’s pH and conductivity values, along with microscopic observation of its colloidal structure, is a straightforward yet valuable technique able to reveal certain aspects of its microstructure [43]. A closer observation of the captured micrographs implied that both investigated emollients were finely and homogeneously dispersed using either 2% (Figure 3a,e) or 3% of the emulsifier (Figure 3c,g). In the applied emulsifier:emollient ratios, the emulsifier appears to be situated mainly on the droplet surface [44]. In the case of Emogreen^®^ L15 emollient, unlike L19, the emulsifier concentration increase has initially led to a certain decrease in droplet size. It is another confirmation of this emollient’s higher droplet deformability with different formulation and processing factors. Re-evaluation of the samples’ microstructures after 3 months of storage failed to discern common emulsion instability issues, implying their preliminary stability.

As presented in Table 1, the pH values of all developed model emulsions were in the narrow range of 6.3–6.4, whereas electrical conductivity ranged from 180 to 625 µS cm^−1^. The initially obtained values endured only subtle changes in the 3-month span, suggesting satisfactory preliminary stability: the pH values remained well within the acceptable ± 0.5 pH units range, while the conductivity values changed no more than 4.32 ± 3.10 µS cm^−1^. According to the ANOVA results (Table 2), the reduced quadratic model generated for pH values did not seem good at prediction (the predicted R² was not as close to the adjusted R², i.e., the difference was more than 0.2). Such a finding was expected considering the very subtle differences in pH values of the investigated model emulsions; in other words, the significant quadratic effect of emollient concentration alone (B2) and its combined effect with emollient type (BC) on emulsion pH value (Table 2) actually had no practical importance. On the contrary, the electrical conductivity of the developed emulsions was significantly dependent on emulsifier concentration (A) and emollient concentration (B) in a linear fashion, as shown in Table 2 and Figure 1f, with emollient concentration having the strongest effect. A negative sign for the coefficient of emollient content represented an antagonistic effect on electrical conductivity, meaning that the electrical conductivity of the prepared emulsions decreased by increasing emollient concentration in the formulation (from 10% to 30%). Conversely, a higher emulsifier concentration (2% vs. 3%) tended to produce emulsions with higher electrical conductivity values.

### 3.4. Formulation Optimization

After analyzing the effects of the investigated formulation variables on the physicochemical properties of the developed model emulsions, their simultaneous numerical optimization was performed as a part of the experimental-design study. The ultimate objective was finding a combination of the controllable factors resulting in emulsions with desirable/acceptable rheological properties according to the defined optimality criteria, and, finally, with desired/adequate quality, stability and sensorial performances. During the process of optimization, the desired goals for each tested factor and response were set (emulsifier concentration: 2–3%; emollient concentration: 10–30%; emollient type: Emogreen^®^ L15 or Emogreen^®^ L19; yield point: maximize; and apparent viscosity, hysteresis area and storage and loss moduli: in obtained experimental range) and combined into an overall desirability function, whereby a global desirability value ranging from 0 to 1 was calculated [14,15]. From suggested numerical solutions, the highest yield point along with satisfied apparent viscosity, hysteresis loop area and G′ and G″ values could be obtained with the following factors’ combinations: 2% emulsifier/30% emollient/ Emogreen^®^ L15 or Emogreen^®^ L19 (predicted apparent viscosity 230.7 mPa s, hysteresis area 18 Pa s^−1^, G′ 16.2 Pa, G″ 4.4 Pa, yield point 1.7 Pa and desirability value 0.834), as well as 3% emulsifier/30% emollient/Emogreen^®^ L15 or Emogreen^®^ L19 (predicted apparent viscosity 230.7 mPa s, hysteresis area 16.8 Pa s–1, G′ 16.2 Pa, G″ 4.4 Pa, yield point 1.7 Pa and desirability value 0.834). Considering all the rheological and optimization data, as well as the cost of new product development (specifically the cost of the emulsifier), the natural emulsion formulations containing 2% of the emulsifier and 30% of emollient Emogreen^®^ L15 or Emogreen^®^ L19 could be selected as preferred to achieve the targeted rheological profile and further investigated as potential carriers for cosmetic actives. Having in mind the initially recommended concentration ranges of 0.5–50% for the emollients and 1–3% for the mixed emulsifier, these findings lead to a more rational formulation design space.

### 3.5. Texture, Sensory and Friction Analysis

The performed DoE significantly narrowed down the number of samples about to enter the second stage of purely instrumental (texture) and in vivo studies (friction and sensory). Hence, the following samples will be further discussed: FFE2, FFE9, FFE14 and FFE16, all comprising 30% of a respective Emogreen emollient and either 2% or 3% of the emulsifier. Taking into consideration that sensory and textural properties most frequently determine a product’s consumer acceptance [25], key texture parameters codependent on sensory ones were assessed using two different measuring probes: a cylinder and a cone. Both probes were of the same material in order to be able to assume similar surface energies and magnitude of forces [6].

**Figure 4:**
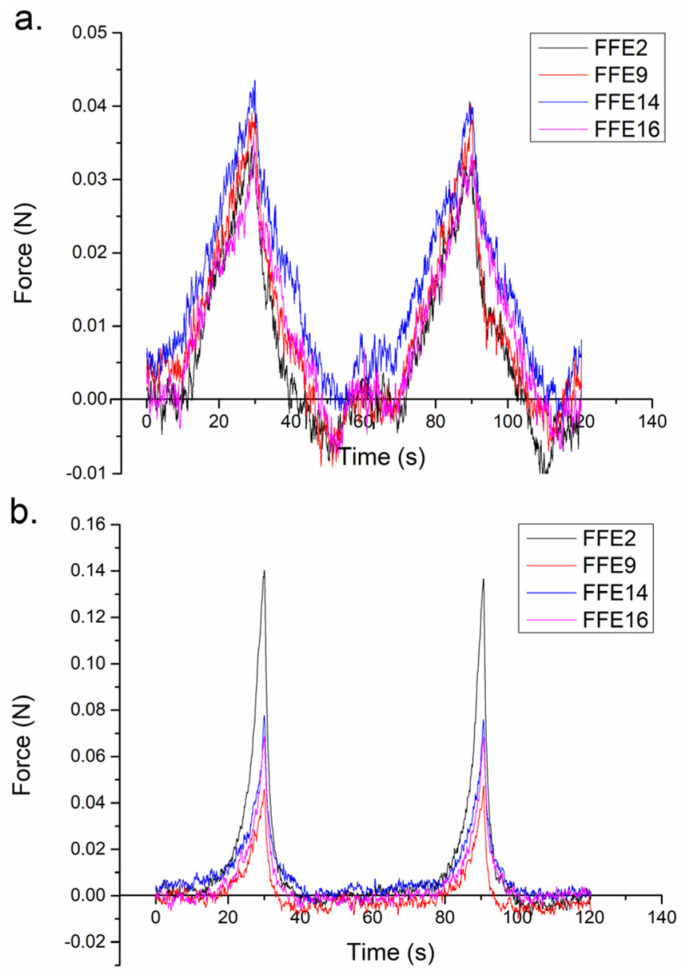
Comparative representation of the selected FFE samples’ texture profiles, using a (**a**) cylindrical measuring probe and (**b**) cone-shaped probe.

Although a comparative view of the obtained texture profiles fails to depict significant changes among the samples (Figure 4a,b), individual analysis of firmness (taken as the maximum value on the texture profile’s positive curve), consistency (area under the positive curve) and cohesiveness (peak value of the negative curve) provided more information. Furthermore, the negative parts of the profile commonly reflect a sample’s adhesiveness. Interestingly, while using the cylindrical measuring probe, sample FFE14 comprising 2% emulsifier and 30% Emogreen^®^ L15 showed significantly higher firmness (42.12 ± 1.56 mN) when compared to FFE2 also stabilized with 2% of the emulsifier but containing 30% of Emogreen^®^ L19 (33.62 ± 2.09 mN). This finding was not expected, considering the inherent viscosity values of these natural emollients (4.5 mPa s for Emogreen^®^ L15 and 9 mPa s for Emogreen^®^ L19), but can again be attributed to highly deformable droplets of the Emogreen emollients when dispersed, as discussed in Section 3.2. In turn, the conical geometry (Figure 3b) revealed the difference between the firmness of the samples stabilized with different emulsifier concentrations (e.g., FFE2 and FFE9). Unexpectedly, the conical probe failed to accentuate the negative parts of the texture profiles but offered a smoother appearance of the negative part of the curve, often held as a desirable quality in topical formulations [45]. Although the investigated emollients were not expected to significantly contribute to a sample’s adhesiveness, Figure 3a discerned sample FFE2 as the most cohesive one. When reassessed after three months of storage at room temperature, no significant changes in texture profiles were observed (data not shown). Therefore, looking at the whole set of the obtained texture analysis results, it can be said that the investigated natural-origin ingredients possess desirable texture profiles [21], robust enough to allow for targeted modifications towards either better spreadability (e.g., sample FFE14) or substantivity (e.g., FFE2 and FFE9).

**Figure 5:**
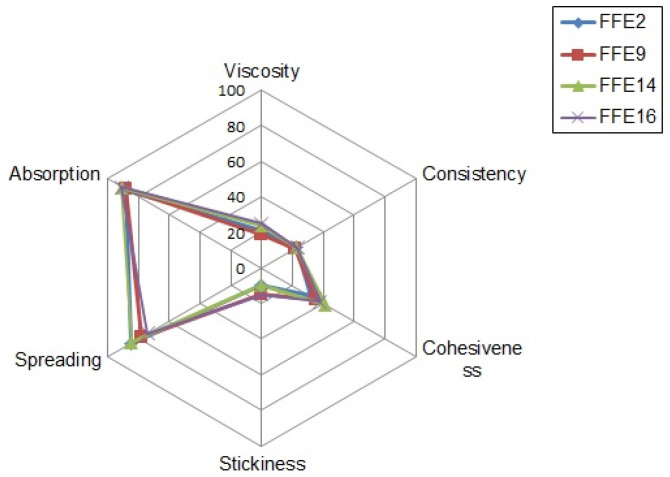
Mean scores of the selected sensory descriptors for the samples FFE2, FFE9, FFE14 and FFE16.

Since all the samples were attributed with very similar objective organoleptic properties (white, moderately shiny and low-viscosity lotions), it was somewhat expected that the panelists would fail to discern statistically significant differences in the following sensory descriptors: viscosity, absorption, residual film and skin hydration (all *p* > 0.05). However, when evaluating spreadability (Figure 5), samples FFE2 and FFE14 were given somewhat higher scores (in the range 85–90 out of 100) than samples FFE9 and FFE16 (75–80 out of 100). This is a direct result of emulsifier concentration variation, having in mind that FFE2 and FFE14 were stabilized with 2% of the emulsifier. The same discrepancy was noted for sample stickiness, since samples stabilized with 3% of the emulsifier were attributed with slightly higher scores (*p* > 0.05). Nevertheless, the actual scores were in the range of 10–15, indicating overall low stickiness of the investigated samples (Figure 4). All in all, the obtained sensory feedback was highly satisfactory, considering that it may determine the consumer’s acceptance of topical emulsions [1].

**Figure 6:**
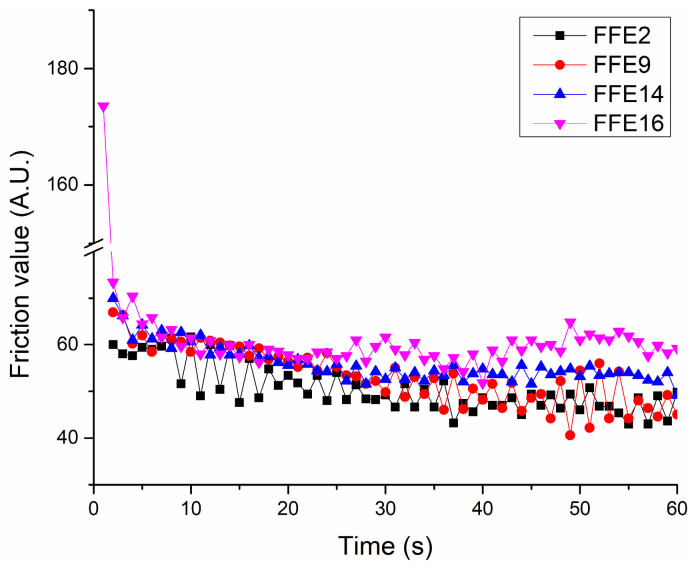
Comparative view of the friction profiles over 60 s measurements mimicking maximum skin application period.

In order to gain better insights into the spreading phenomena on the skin, as well as into the interactions between skin and the residual film formed by the tested emulsions, a tribological study was also performed on human volunteers. All the samples managed to significantly decrease the friction parameters (51 ± 8 for FFE2, 54 ± 7 for FFE9, 57 ± 8 for FFE14 and 62 ± 14 for FFE16) when compared to untreated skin (346 ± 88 for the nontreated control sites; *p* < 0.05). Moreover, looking at the comparative friction profiles (Figure 6), the friction values were higher immediately after the probe application on the skin surface and within a few seconds reached a plateau value for all tested samples (except for FFE9 formulation). This stable pattern of the friction profile [46] implies the formation of a slippery film at the skin surface and a good lubricating effect, hence suggesting a range of possible applications of emulsions based on the investigated emollients (e.g., from prospective skin care to makeup products). In general, longer plateau values indicate a longer lubricating effect of the formulation [28]. This was somewhat expected, knowing that the plateau duration tends to be relatively short for emulsions with polar and semisolid emollients, while it is prolonged in the case of nonpolar and liquid emollients [28]. In this regard, it was interesting to note that sample FFE2 managed to maintain stable and the lowest friction throughout the 60 s measurement period, while sample FFE9 released its full lubricating potential in the second part of the measurement (30–60 s). Both of these samples contained 30% of Emogreen^®^ L19 but differed in the amount of the emulsifier. On other hand, in the case of FFE16 formulation, prepared with 3.0% of the emulsifier and 30% of Emogreen^®^ L15, after the initial plateau (during 25 s), a slight but gradual increase in the mean friction value was observed. This might indicate that the emulsion started to absorb and penetrate into the stratum corneum during the measurement, leading to a more profound skin hydration effect that overcame the simple lubricating effect [28,47]. Nonetheless, no remarkable difference was found between friction profiles of these four FFE samples (Figure 6), which is in line with the previously discussed texture and sensory results and confirms the high similarity of the two green emollients.

## 4. Conclusions

Combining all the results acquired from continuous rotational and oscillatory tests, it could be seen that the developed model emulsions showed favorable shear-thinning flow behavior (with thixotropy and certain yield point) and character of viscoelastic solids in the LVE range of amplitude sweeps. The performed RSM optimal experimental design study revealed significant individual as well as interaction effects of the tested formulation factors (emulsifier concentration and emollient concentration and type) affecting physicochemical properties, specifically rheological properties (apparent viscosity, hysteresis area, yield point and storage and loss moduli), as critical quality attributes of the developed natural emulsions. From the established design space, the model formulations containing 30% of emollient Emogreen^®^ L15 or Emogreen^®^ L19 and stabilized with 2% of the emulsifier were selected as promising carriers for active substances with respect to rheological behavior (the highest yield point and satisfactory apparent viscosity, hysteresis area and G′ and G″ values).

The present study suggested a model procedure for more rapid emulsion formulation development. From 22 prospective samples stemming from the applied RSM optimal experimental design, further structured instrumental assessment refined the number to four promising formulations. Subsequent assessment of texture, friction and sensory studies revealed strengths and weaknesses of each of the four formulations, thus indicating more specific possible applications of each formulation. This is an important asset, especially in the early formulation development stages.

## Figures and Tables

**Table 1:** RSM optimal design matrix with the response values of the developed natural emulsions.

Formulation Code	Factor			Response (Mean ± Standard Deviation)						
	A: Emulsifier Concentration (%, *w*/*w*)	B: Emollient Concentration (%, *w*/*w*)	C: Emollient Type	Apparent Viscosity (mPa s)	Hysteresis Area (Pa s^−1^)	G′ (Pa)	G″ (Pa)	Yield Point (Pa)	pH Value	Electrical Conductivity (µS cm^−1^)
FFE1	3 (+1)	20 (0)	Light liquid paraffin (+1, 0)	167.32 ± 0.37	11.01 ± 0.23	10.20	3.23	1.51	6.35 ± 0.03	314 ± 5.1
FFE2	2 (−1)	30 (+1)	Emogreen L19 (−1, −1)	253.76 ± 1.37	17.68 ± 0.31	18.80	4.79	2.12	6.33 ± 0.08	198 ± 1.7
FFE3	3 (+1)	10 (−1)	Emogreen L15 (0, +1)	154.92 ± 0.19	6.24 ± 0.10	9.62	2.98	1.76	6.39 ± 0.04	389 ± 9.5
FFE4	2 (−1)	20 (0)	Light liquid paraffin (+1, 0)	162.38 ± 1.82	11.51 ± 3.04	10.70	3.23	1.58	6.36 ± 0.06	401 ± 3.6
FFE5	3 (+1)	10 (−1)	Emogreen L19 (−1, −1)	118.88 ± 0.18	8.06 ± 0.04	6.25	2.27	1.23	6.35 ± 0.02	616 ± 6.7
FFE6	2 (−1)	20 (0)	Emogreen L15 (0, +1)	137.41 ± 0.29	5.97 ± 0.79	8.19	2.69	0.67	6.41 ± 0.07	317 ± 3.8
FFE7	3 (+1)	30 (+1)	Light liquid paraffin (+1, 0)	221.57 ± 1.43	17.21 ± 0.15	15.00	4.19	1.24	6.33 ± 0.04	195 ± 4.0
FFE8	2 (−1)	10 (−1)	Emogreen L15 (0, +1)	123.60 ± 0.29	4.35 ± 0.35	7.13	2.41	1.03	6.37 ± 0.08	394 ± 6.1
FFE9	3 (+1)	30 (+1)	Emogreen L19 (−1, −1)	203.77 ± 0.68	15.51 ± 1.50	12.60	3.94	2.05	6.35 ± 0.07	316 ± 4.6
FFE10	2 (−1)	30 (+1)	Light liquid paraffin (+1, 0)	224.55 ± 0.28	16.85 ± 0.82	15.90	4.32	1.80	6.35 ± 0.05	192 ± 5.1
FFE11	2 (−1)	20 (0)	Emogreen L19 (−1, −1)	156.99 ± 0.03	8.72 ± 0.40	9.91	3.16	1.21	6.38 ± 0.03	300 ± 11.0
FFE12	3 (+1)	20 (0)	Emogreen L15 (0, +1)	154.29 ± 0.30	10.65 ± 0.39	9.06	2.94	0.97	6.38 ± 0.06	330 ± 3.8
FFE13	3 (+1)	10 (−1)	Light liquid paraffin (+1, 0)	125.09 ± 0.11	8.57 ± 0.06	6.65	2.37	1.51	6.26 ± 0.03	587 ± 14.0
FFE14	2 (−1)	30 (+1)	Emogreen L15 (0, +1)	260.88 ± 0.11	18.88 ± 0.97	19.70	4.71	2.61	6.30 ± 0.02	205 ± 1.5
FFE15	2 (−1)	10 (−1)	Emogreen L19 (−1, −1)	124.18 ± 0.04	4.69 ± 0.43	6.99	2.43	1.68	6.29 ± 0.03	438 ± 6.7
FFE16	3 (+1)	30 (+1)	Emogreen L15 (0, +1)	241.22 ± 0.93	17.20 ± 0.50	17.10	4.43	1.82	6.31 ± 0.04	255 ± 12.9
FFE17	3 (+1)	20 (0)	Emogreen L19 (−1, −1)	174.93 ± 0.52	11.57 ± 0.07	10.70	3.34	1.20	6.36 ± 0.05	407 ± 5.5
FFE18	2 (−1)	10 (−1)	Light liquid paraffin (+1, 0)	130.96 ± 0.07	5.95 ± 0.15	7.55	2.54	1.24	6.30 ± 0.03	368 ± 1.7
FFE19	2 (−1)	20 (0)	Emogreen L15 (0, +1)	177.19 ± 0.21	10.90 ± 0.30	11.20	3.39	1.71	6.34 ± 0.01	221 ± 15.9
FFE20	3 (+1)	10 (−1)	Emogreen L15 (0, +1)	114.03 ± 0.19	6.69 ± 0.48	5.83	2.15	1.01	6.37 ± 0.03	457 ± 7.4
FFE21	3 (+1)	10 (−1)	Light liquid paraffin (+1, 0)	145.45 ± 0.59	10.51 ± 0.47	8.62	2.72	0.71	6.30 ± 0.02	625 ± 3.5
FFE22	2 (−1)	30 (+1)	Emogreen L15 (0, +1)	209.13 ± 5.63	19.06 ± 1.14	14.30	4.15	1.15	6.37 ± 0.06	180 ± 18.0

Coded levels of factors are shown in parentheses: the coded −1 level corresponded to a lower value, coded 0 level to a middle value and coded +1 level to an upper value of the investigated factor. Other fixed ingredients in each formulation: Xanthan Gum 0.35% (*w*/*w*), Glycerin 3% (*w*/*w*), Ethylhexylglycerin & Phenoxyethanol 1% (*w*/*w*) and purified water up to 100% (*w*/*w*).

**Table 2:** Statistical analysis and coded equations of the generated models for the evaluated emulsion responses.

ANOVA		Response						
		Apparent Viscosity	Hysteresis Area	G′	G″	Yield Point	pH Value	Electrical Conductivity
Model	Sum of Squares	39,244.72	459.93	304.11	13.39	1.11	0.0181	299,016.67
	df	2	4	2	2	1	6	2
	Mean Square	19,622.36	114.98	152.06	6.70	1.11	0.0030	149,508.34
	F value	70.13	50.67	52.02	90.34	6.05	4.12	32.85
	*p*-value	<0.0001	<0.0001	<0.0001	<0.0001	0.0231	0.0122	<0.0001
Lack of Fit	Sum of Squares	2966.72	23.59	34.40	0.9382	1.70	0.0051	68,198.19
	df	15	13	15	15	16	11	15
	Mean Square	197.78	1.81	2.29	0.0625	0.1063	0.0005	4546.55
	F value	0.3368	0.4842	0.4340	0.5320	0.2180	0.3134	0.9947
	p-value	0.9457	0.8559	0.8939	0.8336	0.9883	0.9428	0.5645
Pure Error	Sum of Squares	2349.28	14.99	21.14	0.4703	1.95	0.0059	18,283.00
	df	4	4	4	4	4	4	4
	Mean Square	587.32	3.75	5.28	0.1176	0.4877	0.0015	4570.75
Cor Total	Sum of Squares	44,560.72	498.51	359.65	14.80	4.76	0.0291	385,497.86
	df	21	21	21	21	21	21	21
Fit Statistics	R^2^	0.8807	0.9226	0.8456	0.9048	0.2324	0.6222	0.7757
	Adjusted R^2^	0.8681	0.9044	0.8293	0.8948	0.1940	0.4711	0.7520
	Predicted R^2^	0.8393	0.8790	0.7915	0.8723	0.0616	0.2389	0.7024
	Adeq Precision	16.3609	18.3399	14.0493	18.6756	4.2205	6.0624	13.2807
Coded Equation		= 161.50 + 50.53∗B + 18.67∗B^2^	= 10.13 + 0.5519∗A + 5.47∗B − 1.12∗AB + 1.81∗B^2^	= 9.99 + 4.43∗B + 1.77∗B^2^	= 3.14 + 0.9388∗B + 0.2826∗B^2^	= 1.46 + 0.2719∗B	= 6.37 + 0.0010∗B − 0.0186∗C [1] + 0.0152∗C [2] + 0.0263∗BC [1] − 0.0270∗BC [2] − 0.0314∗B^2^	= 344.58 + 41.09∗A − 124.34∗B

*p* < 0.05 indicates the corresponding model is significant. Adeq Precision measures the signal to noise ratio; a ratio greater than 4 is desirable. A: emulsifier concentration; B: emollient concentration; C: emollient type.

## Data Availability

Not applicable.
